# Transient Trabecular Formation in the Bladder and Delayed Free Air due to Traumatic Intraperitoneal Bladder Rupture

**DOI:** 10.7759/cureus.14520

**Published:** 2021-04-16

**Authors:** Youichi Yanagawa, Yuta Murai, Yoshihiro Kushida, Mutsumi Sakurada

**Affiliations:** 1 Acute Critical Care Medicine, Shizuoka Hospital, Juntendo University, Izunokuni, JPN; 2 Surgery, Shizuoka Hospital, Juntendo University, Izunokuni, JPN

**Keywords:** bladder perforation, free air, trabecular formation, image, bladder

## Abstract

An H-section steel bar that had been set against the wall fell and hit the abdomen and then both legs of a 33-year-old Chinese man. As his vital signs were stable and his chief complaint was leg pain, he was transferred to a local medical facility. After the confirmation of gross hematuria by an indwelling a bladder catheter there, he was transported to our hospital. On arrival, his vital signs were stable. His main complaint was foot pain. He had a scabbing injury at the scrotum and bilateral foot joint deformity. Whole-body computed tomography (CT) from head to toe revealed trabecular formation in the bladder and slight fluid collection in the rectovesical pouch, as well as bilateral fracture-dislocations at the ankles. The urinary tract injury and the fluid collection in the rectovesical pouch were managed conservatively, and the lower limbs were treated tentatively. Follow-up CT on day 3 revealed multiple free air pockets in the intra-abdominal cavity, which was considered to indicate perforation of the duodenal ulcer and treated conservatively. However, he showed abdominal pain on day 7, and repeated CT revealed increased fluid in the intra-abdominal cavity. Urgent laparoscopy showed intact bowels and perforation of the bladder that was closed by suturing. He ultimately obtained a survival outcome. This is the first case of transient trabecular formation in the bladder and delayed free air due to traumatic intraperitoneal bladder rupture. This unique case adds another radiological finding to the list of documented etiologies of traumatic bladder perforation.

## Introduction

Bladder rupture, a relatively rare condition, is most commonly due to abdominal and/or pelvic trauma, but it may be spontaneous or iatrogenic in association with surgical or endoscopic procedures [1-3]. Bladder rupture may occur in the peritoneal space but is more commonly extraperitoneal. Pelvic pain and gross hematuria are present in most patients. The diagnosis is confirmed with retrograde cystography, either with computed tomography (CT) or plain film. After intraperitoneal bladder rupture, urine drains into the abdominal cavity and is resorbed into the systemic circulation, resulting in obvious major electrolyte and metabolic abnormalities [4,5]. Free air due to bladder rupture has been rare, and this situation has been initially treated as bowel perforation.

We herein report a case of free air due to traumatic intraperitoneal bladder rupture initially treated as duodenal perforation.

## Case presentation

An H-section steel bar that had been set against the wall fell and hit the abdomen and then both legs of a 33-year-old Chinese man. He was unable to stand due to his leg injuries and was transferred to a local medical facility. His vital signs were stable and his chief complaint was leg pain. After the confirmation of gross hematuria by indwelling a bladder catheter, he was transported to our hospital because there were no urologists at the local medical facility. He had no specific past or family history.

**Figure 1:**
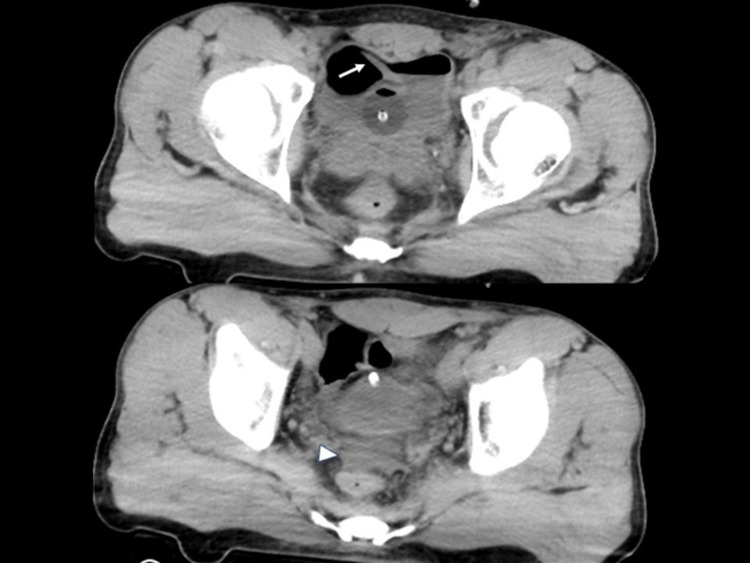
Computed tomography (CT) findings on arrival CT revealed trabecular formation in the bladder (arrow) and slight fluid collection in the rectovesical pouch (triangle).

On arrival, his vital signs were as follows: Glasgow Coma Scale of E4V5M6, blood pressure of 120/63 mmHg, heart rate of 90 beats per minute (BPM), respiratory rate of 20 breaths per minute, and percutaneous saturation of oxygen under room air of 97%. His main complaint was foot pain. He had a scabbing injury at the scrotum without abdominal signs and bilateral foot joint deformity. The results of a biochemical analysis of the blood were as follows: total protein of 6.2 g/dL, albumin of 4.0 g/dL, total bilirubin of 1.2 mg/dL, aspartate aminotransferase of 48 IU/L, alanine aminotransferase of 17 IU/L, lactate dehydrogenase of 294 IU, creatine phosphokinase of 2,374 IU/L, glucose of 110 mg/dL, HbA1C of 5.5%, blood urea nitrogen of 27.1 mg/dL, creatinine of 1.63 mg/dL, uremic acid of 6.5 mg/dL, sodium of 140 mEq/L, potassium of 4.2 mEq/L, calcium of 8.7 mg/dL, C-reactive protein of 0.02 mg/dL, white blood cell count of 23,600/μL, hemoglobin of 14.0 g/dL, platelet of 22.5×10^4^/μL, prothrombin time of 12.4 (11.7) seconds, activated partial thromboplastin time of 26.2 (26.7) seconds, fibrinogen of 185 mg/dL, and fibrin degradation product of 29.1 μg/mL. Whole-body CT from head to toe revealed trabecular formation in the bladder and slight fluid collection in the rectovesical pouch (Figure 1), as well as right fracture-dislocation of the Lisfranc joint and left fracture-dislocation of the ankle.

Initially, he received a diagnosis of urinary tract injury and fracture-dislocations of the bilateral lower limbs.

**Figure 2:**
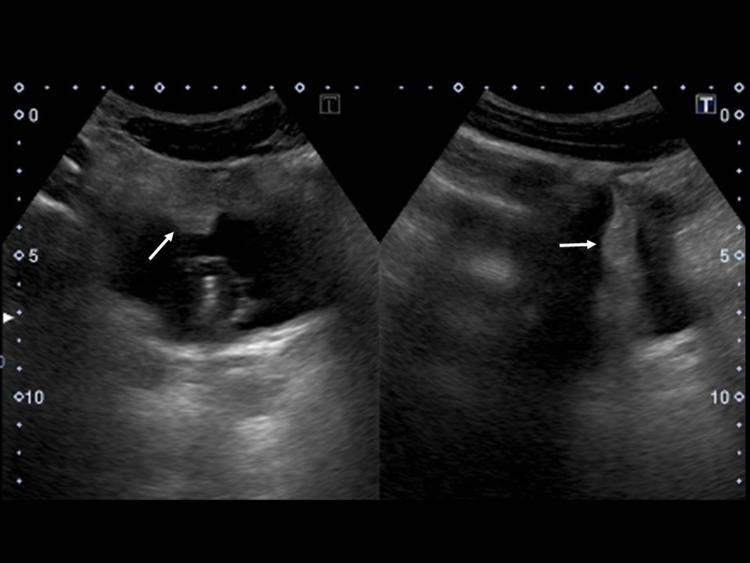
Ultrasound findings on day 2 Ultrasound on day 2 also indicated trabecular formation (arrow) in the bladder.

The urinary tract injury and fluid collection in the rectovesical pouch were managed conservatively with fasting based on the urologists’ decision without further examination, and the lower limbs were treated by external fixation and pinning. Ultrasound on day 2 also indicated trabecular formation in the bladder (Figure 2).

**Figure 3:**
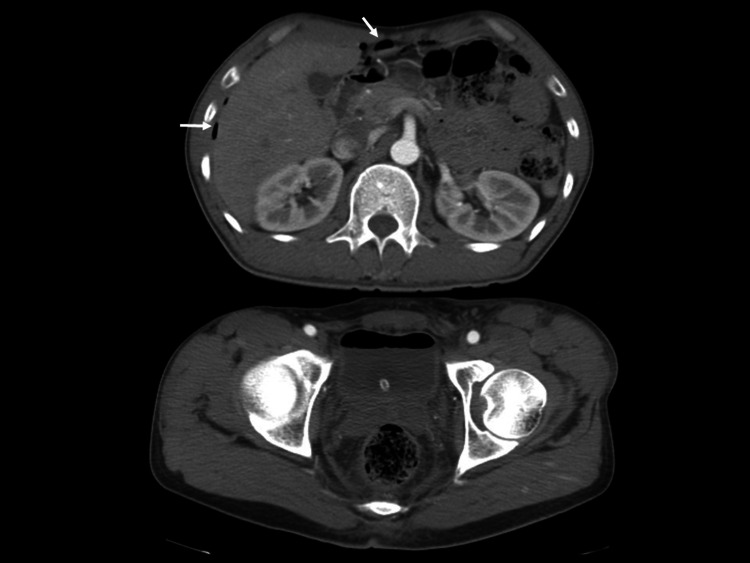
Follow-up computed tomography (CT) findings on day 3 Follow-up CT on day 3 revealed multiple free air pockets in the intra-abdominal cavity (arrow) and the disappearance of the fluid collection and trabecular formation in the bladder.

Follow-up CT on day 3 revealed multiple free air pockets in the intra-abdominal cavity and the disappearance of the fluid collection and trabecular formation in the bladder (Figure 3); however, there were no peritoneal stimulation signs. On the same day, the gross hematuria improved; however, microscopic hematuria remained (50-99 red cells per high-power field).

He underwent emergency gastroscopy, resulting in the discovery of a duodenal ulcer at the bulbus, and therefore the free air was considered to indicate perforation of the duodenal ulcer and was treated conservatively with fasting.

However, he experienced abdominal pain on day 7 with peritoneal stimulation signs, and immediate CT revealed increased fluid in the intra-abdominal cavity. He underwent urgent laparoscopy, which showed intact bowels and perforation of the bladder, which was closed by suturing. From day 8, he started to drink and eat. On day 12, his lower limbs underwent internal fixation. He was then transported to another medical facility for rehabilitation.

## Discussion

To our knowledge, this is the first case to show traumatic transient trabecular formation in the bladder. Initially, we thought this was a congenital abnormality or tumor because the trabecular formation did not move, even with the application of pressure during an ultrasound examination [6,7]. The mobility of clots when a patient changes position is a striking sonographic feature; however, this was different [8]. The first possible explanation for the transient trabecular formation in the bladder is that, like a stalactite or stalagmite in a limestone cavern, bleeding from the injured bladder wall might have induced the formation of a bloody trabecular formation growing down from above and/or building up from below. The second possible explanation is that partial dissection of the inner lining of the ventral bladder wall occurred due to traumatic impact, causing part of the inner lining, which was lined with clotted blood, to hang down inside the bladder. Then dissected inner lining, like a trabecula, was spontaneously cut off due to its deadweight and was drained from the external urethral meatus. To diagnose a transient trabecular formation correctly, urgent retrograde cystoscopy may be helpful when such an image is obtained after traumatic impact.

The bladder rupture in the present case was initially missed, though urologists were consulted. The present case did not have pelvic fracture, which is a common cause of blunt traumatic bladder rupture, and the main complaint was foot pain; however, an indwelling bladder catheter revealed gross hematuria, and fluid collection was noted in the rectovesical pouch on initial CT. In addition, our patient showed azotemia and increasing serum creatinine levels. Ascites and increasing serum creatinine levels are often noted in patients with acute kidney injury. However, these findings are also observed in patients with intraperitoneal urinary leakage [9]. Again, retrograde cystography could have been performed on day 1 to confirm the presence of bladder perforation.

This case showed delayed free air, and the free air was initially considered to indicate perforation of a duodenal ulcer. Free air induced by traumatic bladder perforation is an extremely rare finding, and previous reports have also initially treated such cases as bowel perforation [4,5]. However, this case had gross hematuria, fluid collection in the rectovesical pouch, and azotemia, and increasing serum creatinine levels were confirmed in the initial examination. Accordingly, free air with signs of bladder rupture should raise the suspicion that the free air is induced by bladder rupture. Laparoscopy instead of gastroscopy might be the ideal management in the present case. Uncomplicated extraperitoneal rupture can be managed non-operatively with a catheter, while intraperitoneal rupture requires surgical repair. A delayed diagnosis of bladder perforation might result in a fatal outcome. Fortunately, the present patient survived.

## Conclusions

This was a case of transient trabecular formation in the bladder and delayed free air due to traumatic intraperitoneal bladder rupture. This unique case adds another radiological finding to the list of documented etiologies of traumatic bladder perforation.

## References

[1] Simon LV, Sajjad H, Lopez RA, Burns B (2021). Bladder rupture. StatPearls [Internet].

[2] Trinci M, Cirimele V, Cozzi D, Galluzzo M, Miele V (2020). Diagnostic accuracy of pneumo-CT-cystography in the detection of bladder rupture in patients with blunt pelvic trauma. Radiol Med.

[3] Guttmann I, Kerr HA (2013). Blunt bladder injury. Clin Sports Med.

[4] Merguerian PA, Erturk E, Hulbert WC Jr, Davis RS, May A, Cockett AT (1985). Peritonitis and abdominal free air due tointraperitoneal bladder perforation associated with indwelling urethral catheter drainage. J Urol.

[5] Félix M, Lopes MF (1997). Pneumoperitoneum in bladder rupture. Acta Med Port.

[6] Crisan N, Andras I, Coman I (2014). Editorial comment on: A. Smereczyński, T. Szopiński, T. Gołąbek, O. Ostasz and S. Bojko Sonography of tumors and tumor-like lesions that mimic carcinoma of the urinary bladder. J Ultrason.

[7] Egghart G, de Petriconi R, Söndgen W, Peiberg G (1984). Bladder duplication. A case report. Urologe A.

[8] Jain A, Mahato A, Jacob MJ (2018). Hematoma in urinary bladder masquerading as neoplastic mass. Indian J Nucl Med.

[9] Goto S, Yamadori M, Igaki N, Kim JI, Fukagawa M (2010). Pseudo-azotaemia due to intraperitoneal urine leakage: a report of two cases. NDT Plus.

